# The twisted pharynx phenotype in *C. elegans*

**DOI:** 10.1186/1471-213X-7-61

**Published:** 2007-06-01

**Authors:** Claes Axäng, Manish Rauthan, David H Hall, Marc Pilon

**Affiliations:** 1 Dept. of Chemical and Biological Engineering, Lundberg Laboratory, Chalmers University, Box 462, S-405 30, Göteborg, Sweden; 2 Dept. Neuroscience, Albert Einstein College of Medicine, Bronx, New York, NY 10461, USA; 3 Dept. Cell and Molecular Biology, Göteborg University, Box 462, S-405 30, Göteborg, Sweden

## Abstract

**Background:**

The pharynx of *C. elegans *is an epithelial tube whose development has been compared to that of the embryonic heart and the kidney and hence serves as an interesting model for organ development. Several *C. elegans *mutants have been reported to exhibit a twisted pharynx phenotype but no careful studies have been made to directly address this phenomenon. In this study, the twisting mutants *dig-1*, *mig-4*, *mnm-4 and unc-61 *are examined in detail and the nature of the twist is investigated.

**Results:**

We find that the twisting phenotype worsens throughout larval development, that in most mutants the pharynx retains its twist when dissected away from the worm body, and that double mutants between *mnm-4 *and mutants with thickened pharyngeal domains (*pha-2 *and *sma-1*) have less twisting in these regions. We also describe the ultrastructure of pharyngeal tendinous organs that connect the pharyngeal basal lamina to that of the body wall, and show that these are pulled into a spiral orientation by twisted pharynges. Within twisted pharynges, actin filaments also show twisting and are longer than in controls. In a mini screen of adhesionmolecule mutants, we also identified one more twisting pharynx mutant, *sax-7*.

**Conclusion:**

Defects in pharyngeal cytoskeleton length or its anchor points to the extracellular matrix are proposed as the actual source of the twisting force. The twisted pharynx is a useful and easy-to-score phenotype for genes required in extracellular adhesion or organ attachment, and perhaps forgenes required for cytoskeleton regulation.

## Background

The pharynx is a simple muscular epithelial tube responsible for the ingestion and maceration of food in *C. elegans*. There are 80 cell nuclei from five cell types present in the pharynx: muscle cells, nerve cells, marginal cells, epithelial cells and gland cells [1,2]. The pharynx is sometimes considered to be evolutionarily related to the heart because: (i) like the heart, the pharynx is a rhythmically contracting muscular pump [3]; (ii) the muscle cells of the pharynx have autonomous contractile activity reminiscent of cardiac myocytes [4]; and (iii) *ceh-22*, the *C. elegans *homolog to the homeobox gene Nk2x.5 that plays an important role in heart development in vertebrates, participates in pharyngeal development [5]. Parallels between pharyngeal development and mammalian kidney tubulogenesis have also been made based on the similarity of the cellular processes by which these biological tubes form *de novo *through epithelialization and cell polarity rearrangements [6].

Several *C. elegans *mutants have been reported to exhibit a twisted pharynx phenotype. Two of these mutants, *dig-1 *and *mig-4*, were isolated in screens for mutants with defects in the shape of the hermaphrodite gonad and likely reflect migration defects by the distal tipcells [7,8]. The molecular identity of *mig-4 *is unknown. *dig-1 *corresponds to the predicted gene K07E12.1a/b, which encodes a large protein of 13 100 amino acids containing several EGF-like domains, immunoglobulin domains, von Willebrand factor type A domains, Fibronectin type III domains, and one Sushi domain (SCR repeat) [9,10]. DIG-1 likely straddles the basement membrane mediating specific contacts between cells and their environments.

The uncoordinated mutant *unc-61 *also has a twisted pharynx. *unc-61 *encodes a septin, that is a GTPase required for cytokinesis and other processes requiring spatial organization of the cell cortex, and which can regulate actin dynamics as well as form filamentous polymers. Interestingly, a mutation in the other *C. elegans *septin, *unc-59*, does not cause a twisted pharynx; this is thus one of thefew phenotypes for which *unc-59 *and *unc-61 *differ [11].

Finally, the *mnm-4 *mutant was isolated in a screen for abnormal morphology of the M2 pharyngeal neurons, and also has a twisted pharynx [12]. The molecular identity of *mnm-4 *is still unknown.

The aim of the present study was to better understand the twisted pharynx phenotype and its relationship to extracellular matrix components. Our observations suggest that defects at actin anchorage points to the ECM during larval growth cause the progressive twist to develop intrinsic to the organ.

## Results

### Quantitation of the twisted pharynx phenotype

**Figure 1:**
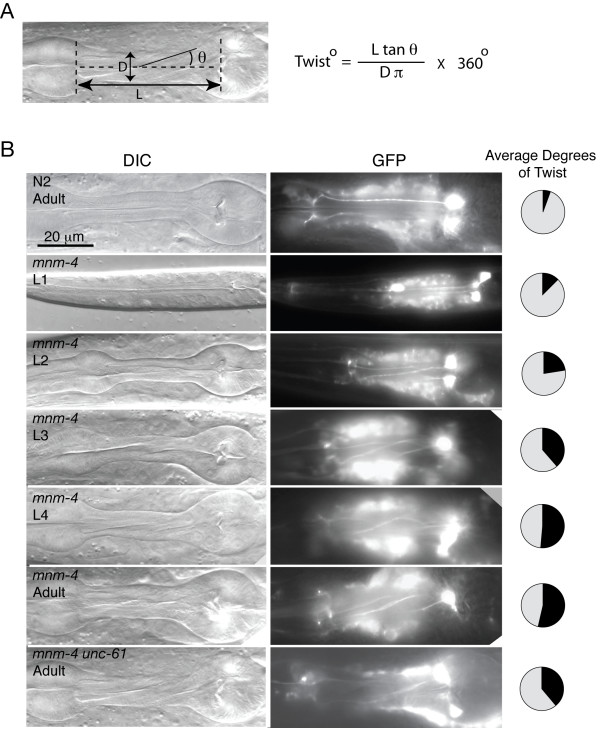
**The twisted pharynx phenotype in *mnm-4;etIs2 *worms of different stages, and in adult *mnm-4;unc-61***. (A) An example of a twisted pharynx and the three measurements that can be obtained by DIC microscopy to estimate the actual degree of twist within the isthmus using the formula shown on the right side (D is diameter; L is isthmus length; and θ is the angle between the torsion lines and the pharyngeal axis). (B) Analysis of *etIs2 [pRF4 pRIC-19::GFP] *transgenic worms [12]: DIC images (left column), M2 neurons of the same worms visualized via their GFP expression (middle column), and the average degree of twist within the isthmus for at least three similar worms scored using confocal microscopy (pie charts). Genotypes and stages are as indicated in the DIC images. See Table 1 for actual numerical data and list of alleles. For the confocal microscopy analysis, worms were mounted on dried agarose pads (2% in dH_2_O), paralyzed with a small drop of 100 mM levamisole and covered with a coverslip. The worms were examined using a Zeiss LSM 510 META system connected to an inverted Zeiss Axiovert 200 microscope. The z-stacks were projected in 360° using 32 or 64 steps and then exported as full resolution images in avi or mov format using the in-microscope software LSM 510 ConfoCor2 Combination, version 3.2. These movies were then used to determine the degree of twisting in the isthmus using video editing software (Sorenson squeeze, trial version). "180° twist" means that the distal ends of the M2 neurons would have to be rotated by 180° in order to be parallel with the cell bodies from which they originate.

By measuring the torsion lines seen in a twisted pharynx, one can calculate the degree of twist (Fig. 1A). This is useful because it allows us to quantitatively measure pharyngeal twisting, even in live animals as they mature. We verified the validity of this mathematical relationship using 3D reconstructions obtained by confocal microscopy of pharynges that expressed GFP in the M2 pharyngeal neurons (Fig. 1B, Table 1; see also Additional files 1 and 2). Note that the degree of twist in the examined mutants was the same between non-transgenic worms and worms in which *rol-6 (su1006dm) *or *dpy-20 (+) *were used as phenotypic markers to establish the transgenic lines (Table 1): these markers therefore do not in themselves affect intrinsic pharyngeal twisting.

**Table 1:** Degrees of twist within the isthmus in animals of various ages and genotypes.

**Genotype, Stage**	**Twist° in Individuals**	**Avg + SEM of |Twist|**
**Samples quantitated from 3D reconstructions**		

*etIs2*, Adult (1d)	17, 6, 23, 36	21 ± 6
*etIs2;mnm-4*, L1	54, 36, 45	45 ± 5
*etIs2;mnm-4*, L2	90, 72, 90, 90	86 ± 5
*etIs2;mnm-4*, L3	135, 107, 152, 163	139 ± 12
*etIs2;mnm-4*, L4	216, 190, 198, 198, 201	201 ± 4
*etIs2;mnm-4*, Adult (1d)	180, 198, 198, 198	194 ± 5
*etIs2;mnm-4*, Adult (6d)	180, 216, 144, 216, 189	189 ± 13
*etEx2;mnm-4*, Adult	220, 210, 186, 231	212 ± 10
*etIs2;dig-1*, Adult	+40, 169, +242, +163	154 ± 42
*etEx2;dig-1*, Adult	170, +90, 45, 62	102 ± 32
*etIs2;unc-61*, Adult	135, 129, 79, 90	114 ± 15
*etEx3;mnm-4 unc-61*, Adult	129, 146, 146, 141	141 ± 4

**Samples quantitated from photographs**		

*etIs2*	0, 0, 0, 0	0
*etIs2;mnm-4*, L4	229, 264, 210	234 ± 16
*mnm-4*, L4	220, 169, 244	211 ± 22
*etIs2/+;mnm-4/+*, L4	79, 71, 71	74 ± 3
*mig-4, Adult	+106, +57, +166, 114, 95, 92	99 ± 14
*mig-4;mnm-4*, Adult	159, 172, 130	154 ± 12
*dig-1, Adult	+107, +46, +107, 108, 141	105 ± 15
*dig-1;mnm-4*, Adult	209, 109, 214	177 ± 34
*eat-3;mnm-4*, L4	119, 89, 123, 89, 126, 130, 85	109 ± 8
*etIs2;mnm-4*, early L2	0, 67, 90, 105	66 ± 23

**Samples quantitated from photographs of the same four developing individuals**		

*etIs2;mnm-4*, late L2	131, 162, 150, 67	128 ± 21
*etIs2;mnm-4*, early L3	214, 157, 173, 144	172 ± 15
*etIs2;mnm-4*, late L3	217, 203, 140, 183	186 ± 17
*etIs2;mnm-4*, early L4	203, 186, 187, 204	195 ± 5

Twisting was measured from 3D reconstructions of worms expressing *pRIC-19::GFP *in their M2 neurons, or by applying the equation shown in Fig 1A on photographs. Besides the *pRIC-19::GFP *plasmid, the *etIs2 *integrated array and the *etEx2 *extrachromosomal array also carry the *rol-6 *transformation marker while the *etEx2 *extrachromosomal array carries the *dpy-20(+) *marker (see Materials and Methods). Most twisting pharynges twisted to the left; right-handed twists are indicated with plus signs ("+"). The *dig-1 *and *mig-4 *worms fell into two groups: those with no pharyngeal twist, and those with an obvious twist. 19 of 24 *dig-1 *and 18 of 24 *mig-4 *adult animals scored did not show any pharyngeal twisting. *Only those *dig-1 *and *mig-4 *worms with a pharyngeal were used to calculate the average twist of these mutants.

### Properties of the twisted pharynx phenotype

We began by studying the *mnm-4 *mutant and found that its pharyngeal twist is first visible post-embryonically and increases during development throughout the larval stages, rather than only in sudden jumps during molting, to reach its maximum during the fourth larval stage (Fig. 1; Table 1). Worms heterozygous for the *mnm-4 *mutation also exhibited a twisted pharynx, although the twist was less pronounced than in age-matched homozygous mutants (Table 1). Furthermore, we noted that *mnm-4 *always makes a left-handed twist, (i.e. a counter-clockwise turn of the nose end if one imagines looking at the worm along its anterior-posterior axis with the nose at the far end). We then quantitated the pharyngeal twisting and handedness also for the *unc-61*, *dig-1*, and *mig-4 *mutants (Table 1 and Fig. 1) and found that all *mnm-4 *and *unc-61 *animals have a left-handed pharyngeal twist, while 25% of *dig-1 *and *mig-4 *animals have either a left- or right-handed pharyngeal twist.

The double mutants *mnm-4 unc-61 *and *dig-1;mnm-4 *showed intermediate twisting phenotypes compared to the single mutants in the pair, and always twisted to the left (Table 1 and Fig. 1). This result suggests that these three genes may either interact directly or are involved in multiple steps in a single developmental process during larval growth of the pharynx. Also, it is clear that the *mnm-4 *left-handed twist isdominant over the ambivalence in twisting direction of the *dig-1 *mutant, which tentatively places *mnm-4 *downstream of *dig-1*.

### Pharyngeal twist is retained in isolated pharynges, and thickened pharyngeal parts resist twisting

We were interested to test whether the force causing twisting is an intrinsic property of the organ or originates from outside the pharynx. Tothis end, pharynges where dissected away from the rest of the worm, then allowed to relax in an isotonic medium. They were then scored for the presence and extent of twist.

**Figure 2:**
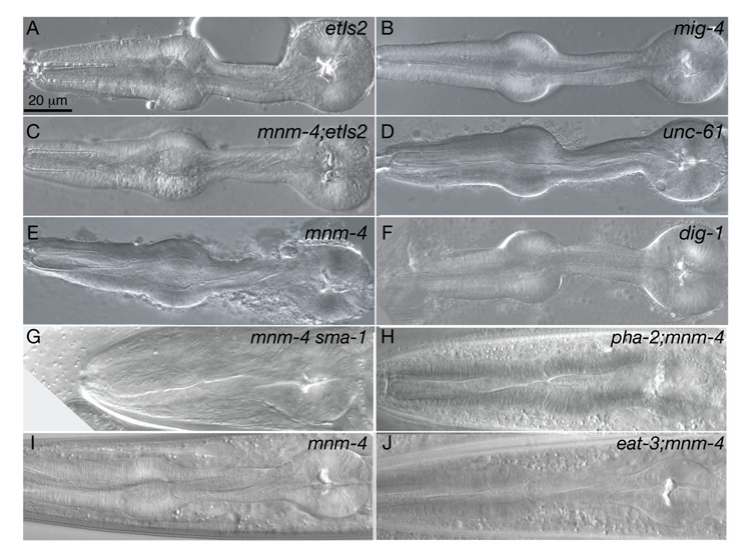
**Description of isolated pharynges and abnormal pharynges**. For panels A-F, pharynges were dissected from worms held on dried agarose pads (2% in H_2_O) covered with mineral oil and using a tungsten (0.5 mm) dissecting needle, then transferred to a drop of M9 on an agarose pad (2% in M9), allowed to relax by swirling the liquid before applying a coverslip, and examined under the microscope. Note that the control pharynx of wild-type worms carrying the *etIs2 *integrated sequence is straight, and that the isolated pharynges of *mnm-4;etIs2*, *mnm-4*, *mig-4 *and *unc-61 *worms are twisted whereas the pharynx of the *dig-1 *animal is untwisted. Panels G-J show the pharyngeal twist in *mnm-4 *animals carrying mutations that cause abnormal pharynx morphologies (*pha-2*, *sma-1) *or reduced pumping rate (*eat-3*). Worms in G and H are adults, and L4 larvae are shown in I-J. Note that the enlarged procorpus of *mnm-4;sma-1 *worms and the enlarged isthmus of *mnm-4; pha-2 *worms show less twisting then the corresponding part of control worms, and that the *mnm-4 *is slightly more twisted than the *eat-3;mnm-4 *worm.

We found that the pharynx of wild-type worms does not twist when dissected away from the body. Conversely, the pharynx of *unc-61*, *mig-4*, *dig-1 and mnm-4 *mutant worms mostly keep their twisted shape after being dissected (Fig. 2A–F). This result does not exclude the possibility that the twist originates from outside the pharynx, but it shows that the twist has become irreversible by the time of dissection. The result is also consistent with the twisting force being intrinsic to the pharynx itself. The pharynx of *dig-1 *and *unc-61 *mutants occasionally straightens (2 of 15 and 3 of 16 dissected pharynges, respectively) when separated from the worm. It is possible that the rare untwisted examples are due to damage to the pharyngeal basal lamina during dissection. We also observed that the thickened pharyngeal regions (procorpus or isthmus) found in the *sma-1 *or *pha-2 *mutants are resistant to the pharyngeal twist normally induced by the *mnm-4 *mutation (Fig. 2G–H). Consistently, the narrower pharyngeal isthmus and procorpus show greater twisting than the pharyngeal bulbs in all alleles.

### The twisted pharynx is fully functional

**Figure 3:**
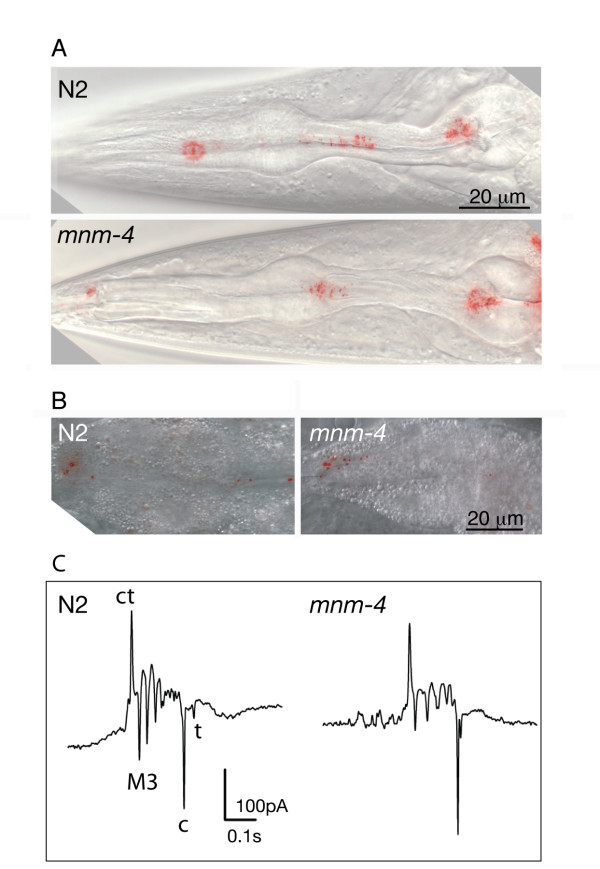
**Comparison of the ability of N2 and *mnm-4 *worms to ingest fluorescent beads**. (A) Overlays of a DIC image and an epifluorescence image of the fluorescent beads present in the pharynx following a feeding period of 30 minutes. Number of beads counted in the pharynx: 64.6 ± 9.3 for N2, and 78.1 ± 11.4 (n = 19), indicating a similar steady-state number of beads in the two genotypes; (B) Overlays of a DIC image and an epifluorescence image of thefluorescent beads in the intestine swallowed during a brief 2 min period. Number of beads in the intestine: 27.1 ± 7.4 for N2, and 30.8 ± 7.7 (n = 35), indicating a similar transfer rate of beads from the pharynx to the intestine in the two genotypes. L4 larvae and adults are shown in (A) and (B), respectively.; (C) Electropharyngeograms in wild-type and *mnm-4 *one day-old adults. Labels in the wild-type recording indicate pharyngeal behaviours that are reflected by the electropharyngeogram, and are based on published studies [13,14]. ct: corpus and terminal bulb contraction; M3: M3 neuron-dependent negative spikes (only the first spike is labeled), c: corpus relaxation; t:terminal bulb relaxation. All the expected activity signals are present in the appropriate proportions in the *mnm-4 *mutant, and the slight differences between the two recordings are well within the variation exhibited by both genotypes.

Twisted pharynges performed as well as wild-type pharynges in bead uptake assays designed to measure feeding: there was a similar steady state number of beads within the pharynx (i.e. beads did not become stuck in twisted pharynges) and a similar rate of transfer to the intestine (Fig. 3). We also saw no obvious difference in growth rate between the genotypes, and thepumping rate also did not differ between the control transgenic strain (*etIs2*; 3.7 ± 0.2 pumps per second), and the strain with the most severely twisted pharynx (*etIs2;mnm-4*; 3.7 ± 0.3 pumps per second). Electropharyngeograms can also be used to monitor the changes in pharyngeal muscle membrane potential and thus assess the normality of each pharyngeal contraction [13,14]. Electropharyngeograms showed no appreciable difference between wild-type worms and *mnm-4 *mutant worms (Fig. 3C). That mutants with twisted pharynges are otherwise indistinguishable from wild-type worms likely explains why this subtle phenotype has mostly gone unnoticed until now.

### Pumping is an unlikely twisting force

*mnm-4 *6 days old adults showed no increased twisting compared to *mnm-4 *1 day old adults, indicating that pumping alone is not enough to increase the pharyngeal twist (Table 1). However, pumping rate may influence pharyngeal twisting: *eat-3;mnm-4 *double mutants have reduced pumping rate and show a slightly reduced twisting compared to age-matched *mnm-4 *mutants (Table 1 and Fig. 2I–J). However, *eat-3;mnm-4 *double mutants also showed a 10% reduction in pharyngeal length ("L" in Fig. 1A) compared with *mnm-4 *mutants, and this may have contributed to the decrease in twist if growth of the pharynx is important. *mnm-4 *L1 larvae kept in M9 and without foodfor three days showed no increased twist compared to newly hatched L1 larvae (data not shown), suggesting that pharyngeal growth may be required for the twisted phenotype to manifest itself. Note that healthy starved L1's have a pumping rate similar to controls (2.41 ± 0.07 pump/seconds and 2.58 ± 0.05 pump/seconds, respectively; p = 0.08, n = 12), and that starvation stimulates pumping [15].

### The pharyngeal tendons are pulled by the twisted pharynges

**Figure 4:**
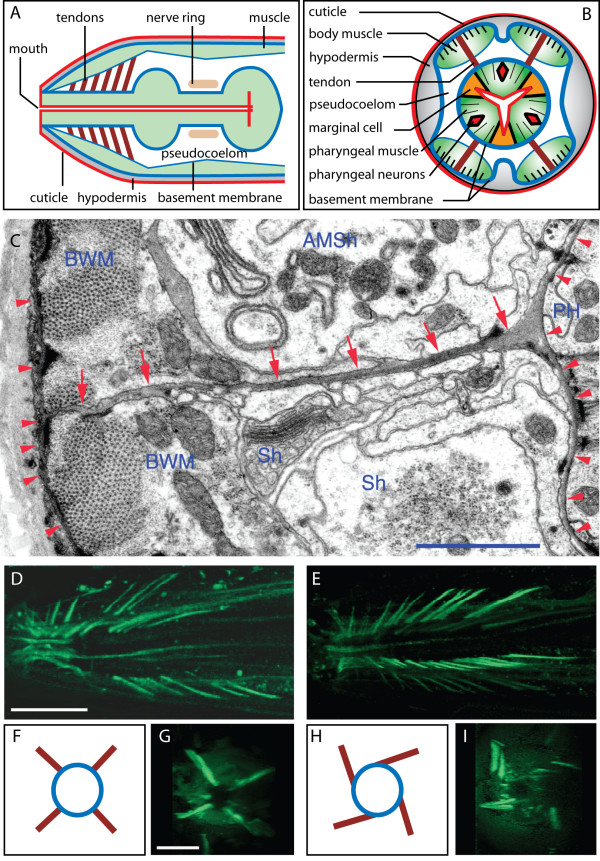
**Anatomical features of the head region and comparison of the hemicentin-rich pharyngeal tendons between wild-type and a mutant with the twisted pharynx phenotype**. (A) and (B) show transverse and cross sections of the head region in idealized forms. For clarity, many structures were omitted here, including axons, excretory canals, muscle arms and complex hypodermal cell shapes that sometimes cover the body muscles. Of particular importance is that the pharynx seems to float in pseudocoelomic fluid and to make almost no contact with the worm body along its entire length: except for the tendons, the pharynx is secured only at its anterior and posterior ends, where it is connected to the mouth and intestine, respectively. (C) Transverse thin section of an adult wild type nose, showing a left ventral tendon (red arrows) connecting the basal laminae (red arrowheads) of the pharyngeal epithelium (PH) and of the body-wall muscles (BWM). Major cells bordering the tendon include the amphid sheath cell (AMSh) and several other sheath cells (Sh) for mechanosensors of the lips. Smaller caliber processes include many sensory dendrites and some arcade processes. Hemidesmosomes link the pharyngeal epithelium's intermediate filaments to the basal lamina. Dense bodies (modified adherens junctions) link the muscle sarcomeres to the muscle's basal lamina. Because it is tilted with respect to the body axis, the tendon is better seen close to the pharynx in this image, but goes out of the plane of section as it passes between the muscle cells. Image is rotated about 20 degrees clockwise for convenience. Scale bar is 1 μm. (D) and (E) show images of a wild-type and *mnm-4 *mutant that carry the hemicentin::GFP transgene *rhIs23 *[30], respectively. (F) and (H): geometry of the tendons (brown) and pharynx (blue circle) viewed in cross sections if the pharynx is not twisted (F) or if it were twisted as a whole while held by the tendons (H). (G) and (I) show cross section views of the flattened confocal image stacks from (D) and (E); note the spiral-oriented tendons in (I). Specimens were immersion fixed using buffered aldehydes and then osmium tetroxide as described previously [40]. Three or four animals were aligned within agar blocks then embedded in plastic resin and sectioned together. Thin cross sections were collected on slot grids, post-stained with uranyl acetate and lead citrate, then examined with a JEOL 1200EX electron microscope. Scale bars in D and H are 10 μm.

The basal surface of the pharynx is covered by a thick basal lamina (See Fig. 4A–C), and four rows of 15–20 acellular tendinous organs composed of hemicentin and fibulin that anchor the anterior end of the pharynx to the basal lamina that covers the bodywall muscles. These tendons, which have also been called "flexible tracks", have been observed in both *C. elegans *and another nematode species [16,17]. They are highly elastic and flexible when observed during normal feeding activity, and we call them "tendons" although we recognize that their attributes are not identical to vertebrate tendons. Because they could be expected to resist pharyngeal twisting, we examined in detail the nature of their attachment using electron microscopy. As shown in Fig. 4C, the pharyngeal tendons are attached to the basal lamina that surrounds the pharynx and project between the bodywall muscle cells in each muscle quadrant, then connect on their opposite ends to the basement membranes running between bodywall muscles and thin extensions of the hypodermal cells, near the cuticle. We used a hemicentin::GFP reporter to visualize the pharyngeal tendons in wild-type and in the *mnm-4 *mutant (Fig. 4D–I). The numbers and thickness of tendons was the same in wild-type and mutant, buttheir orientations in twisted pharynges indicate that they are pulled by the twist, resulting in a spiral-like configuration when viewed along the anterior-posterior axis. Thus the whole pharynx, including its basal lamina to which the tendons are attached, is twisted in mutants. *him-4 *mutants, with defective hemicentin and hence lacking these tendons [2] do not twist, indicating that lack of the support provided by the tendons is not enough to cause a twist. Also, these tendons do not seem to be involved in transferring a twisting force from outside the pharynx since *mnm-4;him-4 *double mutants still have a twisted pharynx (data not shown).

### Twisted pharynges have a subtle actin cytoskeleton defect

**Figure 5:**
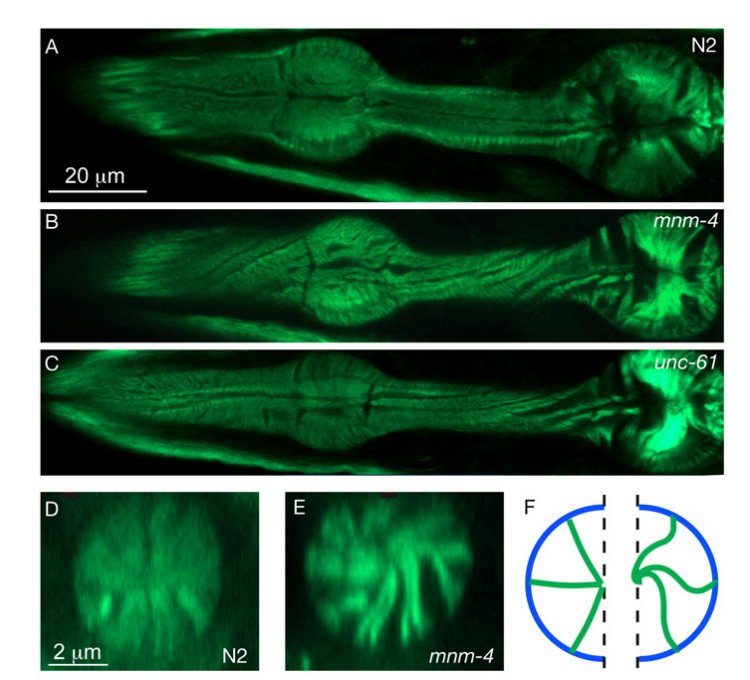
**The actin cytoskeleton is twisted and lengthened in twisted pharynges**. (A-C) Pharyngeal actin was stained using phalloidin-FITC and one confocal section is shown for each of the indicated genotypes. Note that in all the specimens, the actin filaments are evenly spaced and generally aligned near-perpendicularly to the longitudinal axis of the pharynx if it were untwisted. In (B), note that strong degrees of twist can be detected in both the procorpus (leftmost portion) and at the isthmus between the two bulbs in the mutant pharynx, but only subtle twisting within each bulb. (D) and (E) show extracted optical cross sections from within the isthmus of the indicated genotype. The actin fibers are more twisted, hence longer, in the isthmus from *mnm-4 *specimen than in the N2 control. This is illustrated schematically in (F) which depicts the shapes of wild-type actin fibers in the left half, and of *mnm-4 *in the right half. The ratio of the average length of the actin fibers over the isthmus diameter was 0.42 ± 0.02 (s.e.m.) for N2, 0.54 ± 0.03 for *mnm-4*, 0.51 ± 0.04 for *dig-1*, 0.43 ± 0.04 for *mig-4 *and 0.43 ± 0.02 for *unc-61*; n ≥ 3 worms. Only *mnm-4 *significantly differed from N2 (t-test, P = 0.009).

Actin filaments form important radial cytoskeletal structures within the pharyngeal muscles cells [1] and we examined their integrity in twisted pharynges. Using phalloidin as a staining reagent, we found that these actin arrays are significantly longer in *mnm-4 *and, less convincingly, in *dig-1 *mutants than in control worms (Fig. 5). This is interesting since misregulation of filament length in growing pharyngeal muscles may be a cause of twisting: long actin filaments may be accommodated by twisting the pharynx. Alternatively, longer actin filaments could merely be a consequence of the twisted shape.

### The anterior pharynx is firmly anchored to the lips by intercellular junctions, and more tenuously, via buccal cuticle

**Figure 6:**
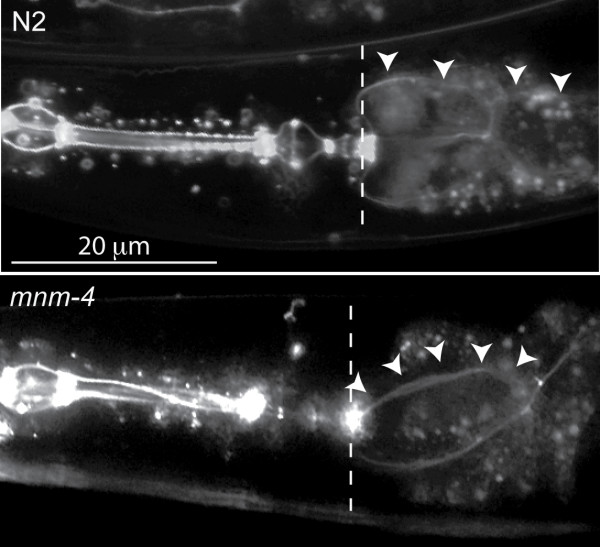
**The anterior part of the intestine is twisted in a direction opposite to that of the pharynx**. Wild-type (N2) and *mnm-4 *mutant adults expressing *ajm-1::gfp *reporter were photographed to reveal not only the twisting within the pharynx of the mutant, but also the opposite twist in its anterior intestine. The dashed line indicates the border between the pharynx (to the left) and intestine. The arrowheads follow a stretch of adherens junctions in the intestine of each animal.

Besides the tendon contacts along its anterior length, the pharynx is alsoconnected directly to the body-wall at its extreme anterior end, at the lips [1]. This connection via the arcade cells is unlikely to allow any local displacement, since it involves many robust intercellular junctions between hypodermis, arcade cells and pharyngeal epithelium. Thus pharyngeal twist must be relative to its fixed orientation at the lips. Indeed, when the twist is visualized using *ajm1:gfp *in the *mnm-4 *background, the pharynx seems to have rotated at the posterior end around the terminal bulb passively dragging the intestine with it, such that the foremost intestinal cells twist in a direction opposite to the pharynx, hence relieving the tension induced by the pharyngeal twist (Fig. 6).

More tenuously, the cuticle lining of the pharynx is also continuous with the bodywall cuticle in the region of the arcade cells (buccal cavity). The body cuticle, buccal cuticle, and pharyngeal cuticle are all shed at each molt (Singh and Sulston, 1978), and the regeneration of new cuticle could contribute to twisting. However, as noted above, we see no sudden changes in pharyngeal twist associated with molting (Table 1). Instead, as the animal grows in size, twisting increases gradually during each larval stage, until it reaches its maximal twist by the L4 stage.

### Direct contact between the basal laminae of the pharynx and body-wall is very tight in the embryo

**Figure 7:**
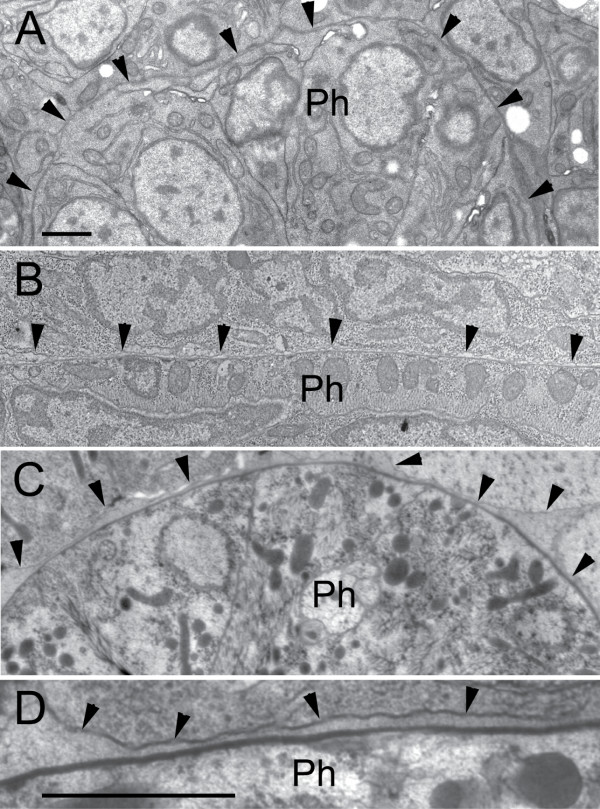
**Basal lamina and pseudocoelom development**. (A) Embryo during morphogenesis, laserhole immersion fixation. Transverse thin section through the head indicates the region between the pharynx (Ph) and bodywall where basal lamina deposition is taking place (arrowheads point to plasma membrane of body-wall ectodermal cells in each panel). During morphogenesis the border between mesoderm and ectoderm is at first highly irregular; in this example the border has become more regular but there is no apparent "pseudocoelomic" space and one cannot readily distinguish separate basal laminae for mesoderm or ectoderm. (B) Embryo just before hatch, chemical immersion fix. Lengthwise thin section of the head shows a slight separation between body-wall cells and the pharyngeal muscles. Radial muscle filaments have developed and the pharynx is now contractile. Virtually no acccessory pseudocoelom is yet observed here, but at higher power the basal laminae of the mesoderm and ectoderm seem to form separate layers by immersion fixation (not shown). (C) Adult head, fixed by HPF and freeze substitution staining. Transverse thin section shows a widened, highly irregular space, the accessory pseudocoelom, has developed between the ectoderm and mesoderm. (D) Higher power view of same region as in C shows the pseudocoelomic space is filled with flocculent matrix materials. The basal lamina of the ectodermal cells cannot be easily distinguished from this flocculent matrix, whereas the very thick basal lamina of the pharynx is now visible as a prominent electron dense line. Pharynx regions are as follows (metacorpus in A; isthmus in B; isthmus in C and D). Scale bars are 1 micron. Scale bar in A is for first three panels.

Multiple studies of *C. elegans *embryos, including our own, have shown practically no available space between the mesodermal layers and ectodermal layers prior to hatching (Fig. 7). In fact there is often no appreciable extracellular space in the head prior to the L1 stage, no matter what fixation is used (chemical immersion fix, microwave fix, or high pressure freezing). Compared to more posterior regions of the body cavity, this tightness remains a feature of the head throughout development, but some minimal separation does develop to accommodate vigorous motions of the pharynx during feeding. Basal lamina deposition can be detected by TEM beginning in mid-embryogenesis using immersion or microwave fixations (Fig. 7A). As shown in Fig. 7B, distinct basal laminae can be seen associated with each tissue in late embryogenesis. Using high pressure freezing, the basal laminae appear much more space filling, and there may be very close contact between the lamina of neighboring tissues. This suggests that appreciable friction between bodywall and pharyngeal tissues may hold the pharynx in place prior to the onset of pumping in the L1, and may thus restrict onset of the twisting phenotype. Only in larvae and adults can a distinct "accessory pseudocoelomic space" be seen that separates the thin ectodermal basal laminae from the thick basal lamina of the pharynx (Fig. 7C–D). It is uncertain whether this accessory pseudocoelom is continuous with the pseudocoelom proper, due to close approach to the pharynx by GLR processes beneath the nerve ring [1,18].

### Unattached pharynges do not twist in L1 larvae

**Figure 8:**
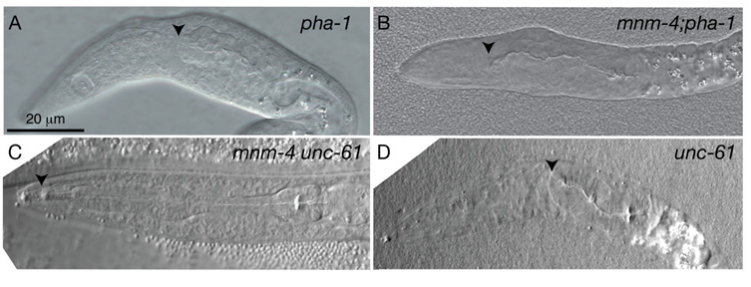
**Anterior detachment of the pharynx does not result in increased or earlier pharyngeal twisting**. Shown are DIC images of L1 larvae of the indicated genotypes and in which the pharynx has detached from the lips and moved posteriorly. Arrows point to the anterior end of the pharynx.

We tried to distinguish between two hypotheses that could explain the increasing twist during larval development. One hypothesis is that the twisting force is already present in early larval stages but that its effect is hampered by attachment of the pharynx to surrounding tissue (see previous section). The other hypothesis is that the increasing twist observed in later larval stages reflects an increase in the twisting force itself, or the cumulative effect of a constant force. Because dissection of pharynges is not practical with young larvae, we studied this using a genetic approach. The *mnm-4 *mutation was introduced into the *pha-1 *genetic background. In *pha-*1 mutants the pharyngeal primordium forms normally and elongates to make contact with the buccal cavity [19,20]. Later during development the connection with the buccal cavity fails at the level of the arcade cells, leading to a Pun (Pharyngeal Unattached) phenotype [19-21]. No twisting could be detected in Pun-pharynges in L1 larvae of either the *pha-1 *single mutant or in the *pha-1;mnm-4 *double mutant (Fig. 8). We conclude that it is not pharyngeal attachment to the lips or surrounding tissues that prevents full twisting in the earliest larval stage, but that a twisting force likely increases, or is constantly applied, during larval development and causes twisting in the mutants.

The *unc-61 *mutant also occasionally shows pharyngeal attachment defects at the L1 stage. Of 57 *unc-61 *L1 larvae scored, 4 had normally developed pharynges that were not attached to the buccal cavity (similar to that shown in Fig. 8C), and 2 had a Pun phenotype (Fig. 8D). Neither of these phenotypes was associated with pharyngeal twisting, againsuggesting that it is not attachment to the lips that suppresses twisting at the L1 stage. *mnm-4 unc-61 *double mutants also showed no increase in the penetration or severity of the *unc-61 *pharyngeal attachment phenotype: of 51 *mnm-4 unc-61 *L1 scored, 4 had pharynges unattached to the buccal cavity (Fig. 8C) and 1 had a Pun phenotype.

### Mini screen of adhesion mutants

Because the pharyngeal twisting phenotype is rather subtle and does not impair feeding, we speculated that perhaps it had been missed during the phenotypic characterization of adhesion molecule mutants. We examined eleven mutations to cause adhesion defects or to affect extracellular matrix molecules [22]. Among these, *sax-7 *was found to exhibit a pharyngeal twisting phenotype with a 33% penetrance (Table 2). The twist in the *sax-7 *mutant was almost always in the leftward direction (27 of 30 twisted pharynges).

**Table 2:** Result of a mini screen of adhesion mutants scored for a pharyngeal twisting phenotype.

**Genotype**	**Allele**	**LG**	**Strain**	**%twisted**	**n**
*adm-4*	*ok265*	X	CZ2744	0	50
*cdh-4*	*ok1323*	III	RB1256	0	40
*ceh-43*	*ok479*	III	RB708	0	47
*egl-27*	*n170*	II	MT170	0	40
*ham-2*	*n1332*	X	MT3149	0	32
*him-4*	*e1266*	X	CB1266	0	38
*ina-1*	*gm39*	III	NG39	0	45
*nid-1*	*cg119*	V	CH119	0	26
*sax-7*	*ok1244*	IV	RB1199	33*	36
*tag-53*	*gk163*	X	VC290	0	50
*zig-6*	*ok723*	X	RB876	0	45

* Of 30 twisting *sax-7 *mutants, 27 twisted to the left, and 3 twisted to the right.

## Discussion

The twisted pharynx is a viable phenotype that worsens during post-embryonic development. Although it can be scored by light microscopy, it has sometimes been overlooked in previous mutant screens. We examined several parameters relevant to understanding the twisted pharynx phenotype. Vigorous pharyngeal motions that accompany molting [23] are not responsible for the twisting force since no sudden increase in twist is produced upon molting (Table 1). This result also suggests that the pharyngeal cuticle is not directly restraining an intrinsic twist. We also found that the attachmentof the pharynx to the bodywall at the lips is not preventing the pharynx from twisting in L1 larvae (Fig. 8), and that the pharyngeal tendons that connect the procorpus to the bodywall appear intact, albeit spirally oriented, in animals with a twisted pharynx (Fig. 4). Twisted pharynges were also functionally indistinguishable from normal pharynges in a bead-uptake assay and pumping rate (Fig. 3). Thus it seems that the twisted pharynx of the studied mutants is functional and properly anchored to the rest of the worm body. Therefore the phenotype likely originates from a relatively minor intrinsic defect within the mutant pharynx. The dissection experiments in which isolated pharynges retained their twisted shape, also show that the twist is an intrinsic property of the pharynges (Fig. 2).

The pharynx features two prominent cytoskeletal filamentous arrays, both of which are oriented in a radial fashion. The marginal epithelial cells have thick bundles of intermediate filaments anchored apically (to cuticle) and basally (to basal lamina) by hemidesmosomes [1]. Since these arrays are not contractile, they likely provide strength and reinforcement to the tissue during pumping, but seem less likely to introduce twisting. Pharyngeal muscles are filled with a space-filling radial array of actomyosin filaments, again anchored to cuticle and basal lamina via specialized actin-based junctions. As the pharynx rhythmically contracts, the actomyosin array foreshortens radially to expand and contract the pharyngeal lumen. This action normally should not introduce any twisting forces, and our observations that the pharynges of 6-day old adult *mnm-4 *mutants twist no more than that of 1-day old adults, and that *mnm-4 *L1 larvae kept in M9 for ~72 hours show no twist indicate that pumping, at least if not accompanied by growth, plays a minor role, if any, in the development of the pharyngeal twist.

Twisting occurs very gradually as the animal and pharynx grows. There is no cell replacement during growth, but each existing pharyngeal muscle mustcontinuously add more actomyosin filaments and reorganize their anchorage points to accommodate growth. The time course of twisting and its steady increase matches this continual process of re-shaping the radial array. Thus the most likely cause of the twisting phenotype is a bias in the repositioning of actin binding sites as that radial array grows. The five mutants with twisted pharynges fell into two categories: the *unc-61 *and *mnm-4 *mutants always made a left-handed twist with 100% penetrance and the *dig-1*, *mig-4 *and *sax-7 *mutants produced a mixture of right- and left-handed twists and with a 25–30% penetrance. Logically, one might expect these mutations to affect proteinsrequired for actin anchorage, either at the basal lamina, or at the pharyngeal cuticle layer. Chiral ordering of these binding sites as they reassemble with respect to their neighbors may account for the observed tendency to generate a left-handed twist in some mutants. This model of pharyngeal twisting being caused by a chiral bias in mispositioning of cytoskeletal elements is mechanistically similar to the reorientation of collagen fibers in Roller mutants, which also shows an allele-specific handedness [24].

Actin filaments normally cycle their subunits via rapid "treadmilling" activity [25]. Interference with normal regulation of actin filament length could produce the observed abnormally long filaments (Fig. 5) and also explain a steadily increasing force that could be relieved by gradualtwisting of the whole pharynx to lengthen each sarcomere. However, this mechanism seems less likely to generate twisting in a favored (lefthanded) direction.

**Figure 9:**
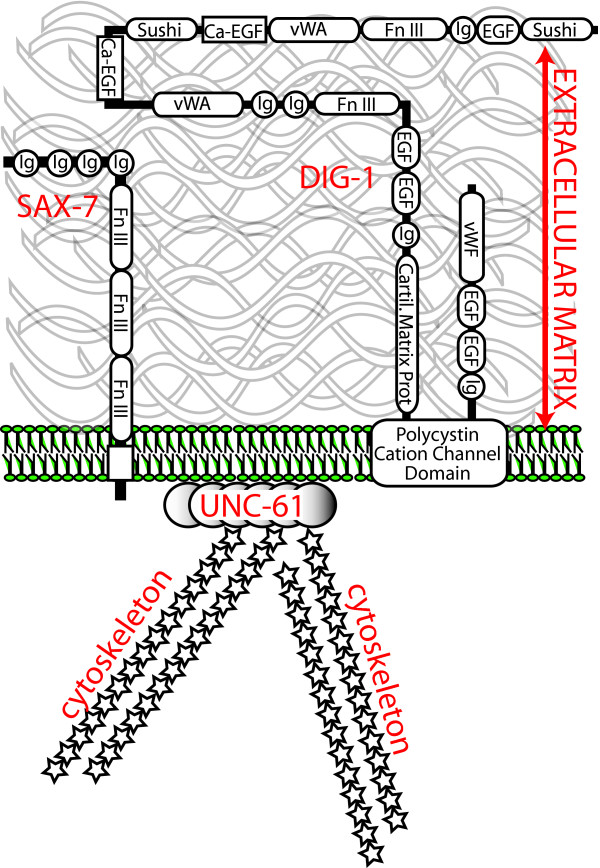
**The DIG-1, SAX-7 and UNC-61 proteins are associated with the plasma membrane**. The diagram describes the localization and general domain structures of the three gene products that are known to cause pharyngeal twisting in mutants and for which the molecular identify is known. This is a speculative model since it has not been demonstrated experimentally that these proteins are co-localized.

The molecular identity of three of the mutations causing a pharyngealtwist is known and is consistent with the phenotype resulting from ECM or cytoskeletal attachment defects (see Fig. 9): the *dig-1 *and *sax-7 *mutations affect adhesion molecules, and *unc-61 *affects a cytoskeleton-organizing septin (the molecular identify of *mig-4 *and *mnm-4 *are unknown). Importantly, all three molecules are expressed in the pharynx [9,26,27].

*dig-1 *encodes a large extracellular matrix adhesion molecule and was originally isolated because of abnormal attachment of the gonad primordium [9,10]. It is likely that its other morphological defects, such as the twisted pharynx, also are caused by impaired adhesion. An interesting observation unrelated to the pharyngeal twist was made when studying the *dig-1;mnm-4 *double mutant: the gonad arms often completely fail to develop, forming instead a small disorganized clear structure in the mid-body region, completely devoid of germ cell nuclei (unpublished observations). This suggests that *mnm-4 *and *dig-1 *have redundant adhesion functions duringgonad development such that the *dig-1 *gonad adhesion defect is worsened in the double mutant. Interactions between cell adhesion molecules and extracellular matrix are known to be important for gonad development[17,28-30].

*unc-61 *is one of two *C. elegans *septins; *unc-59 *is the other. Septins are conserved GTP-binding proteins that polymerize to form filamentous structures that act as scaffolds for membrane- and cytoskeleton-binding proteins [31], a function consistent with our hypothesis that defects in cytoskeleton anchorage points is responsible for the twisted pharynx phenotype. Mutations in the *C. elegans *septins cause defects in distal tip cell and axon migration, as well as in post-embryonic cytokinesis [11,26]. In all of these defects single mutants of either septin gave full penetrance, and double mutants did not exhibit worsened phenotype. However, only *unc-61 *displays the twisted pharynx phenotype. This suggests that their roles in pharyngeal post-embryonic development are independent and distinct from each other. The *unc-61;mnm-4 *double mutant did not show any increase in twisting of the pharynx nor any additive effect on the gonad phenotype (unpublished observations): it is possible that *unc-61 *and *mnm-4 *may function in the same pathway.

The twisted pharynx is a useful and easy-to-score phenotype, allowing one to screen for new genes involved in extracellular adhesion or organ cohesion. Our small pilot screen has already identified one new pharyngeal twisting mutant, *sax-7*, that encodes a cell adhesion molecule important for the attachment of several types of tissues, including neurons, and that, when mutated, causes pleiotropic phenotypes that include uncoordination and embryonic lethality [27,32]. That mutants with twisted pharynges are otherwise indistinguishable from wild-type worms (e.g. they feed normally) likely explains why this subtle phenotype has mostly gone unnoticed.

## Conclusion

1. The twisted pharynx phenotype is found in several *C. elegans *mutants that have defects in adhesion molecules or molecules that regulate attachment of the actin cytoskeleton to the cell cortex.

2. The twisted pharynx phenotype is not a result of pumping activity nor the consequence of an external twisting force applied to the pharynx: it results from a twisting force intrinsic to the pharynx. That force likely results from the defects in the remodeling of the actin attachment points or actin filament length during pharyngeal growth.

## Methods

### Nematode strains and culturing

General methods for the culture, manipulation, and genetics of *C. elegans *were as described [33]. The wild-type parent for all strains was *C. elegans *variety Bristol, strain N2 [34]. All strains were cultured at 20°C (unless otherwise noted) on NGM plates seeded with the *E. coli *strain OP50 [34]. The following mutations and integrated sequences were used in this study:

LGII: *eat-3(ad426)*, *egl-27(n170)*

LG III: *cdh-4(ok1323)*, *ceh-43(ok479)*, *etIs2 [pRF4 pRIC-19::GFP]*, *ina-1(gm39)*, *mig-4*(rh89), *dig-1(n1321)*, *pha-1(e2123)*, *rhIs23 [hemicentin::GFP]*, *Y49E10.20(ok1286)*

LG IV: *dpy-20(e1282)*, *sax-7(ok1244)*

LG V: *mnm-4(et4)*, *nid-1(cg119)*, *sma-1(ru18)*, *unc-61(e228)*

LG X: *adm-4(ok265)*, *ham-2(n1323)*, *him-4(e1266)*, *pha-2(ad472)*, *tag-53(gk163)*, *zig-6(ok723)*

The integrated transgene *jcIs1*, which carries an *ajm-1::gfp *reporter [35], was also used to visualize adherens junctions and was obtained from the *C. elegans *Genetics Center.

### Generation of strains carrying *pRIC-19::GFP*

*C. elegans *transformation was carried out with the use of methodsdescribed by Mello and Fire [36]. Hermaphrodites were injected with a DNA mixture containing 50 μg/m1 plasmid *pRF4*, which carries the transformation marker *rol-6 (su1006dm) *[37], and 50 μg/m1 plasmid *pRIC-19::GFP*, which expresses the RIC-19 protein fused toGFP from the native *ric-19 *promoter [38]. Transgenic lines carrying the microinjected DNA on extrachromosomal arrays were established from F2 Rol progeny of injected animals. In some animals, the plasmid pMH86 containing the wild-type *dpy-20 *gene was used as a transformation marker when injecting *dpy-20 *mutant hermaphrodites; the resulting extrachromosomal array *etEx2 *was then crossed away from the *dpy-20 *background and the transgenic worms identified in later generations by their GFP expression.

### Developmental stage study

For each stage of interest, worms of approximately the right sizes were picked from a mixed age plate while observed under a dissecting microscope. Accurate staging was then established by examining the developmental stage of the gonad and vulva using differential interference contrast (DIC) microscopy at 400× and 1 000× magnification using a Zeiss Axioplan 2 microscope. Staged worms were either scored directly or retrieved and mounted for confocal microscopy. For some studies, worms were mounted just before the expected molting, or just after molting had been observed.

### DIC and Epifluorescent microscopy

For microscopic analysis, worms were mounted on 2% agarose pads, paralyzed with a small drop of 100 mM levamisole, and covered with a coverslip. These were then examined with a Zeiss Axioplan compound microscope using DIC optics or an FITC filter set to visualize GFP. Digital images were acquired using an attached AxioCam digital camera.

### Confocal microscopy

Worms expressing *pRIC-19::GFP *in their M2 neurons were mounted ondried agarose pads (2% in dH_2_O), paralyzed with a small drop of 100 mM levamisole and covered with a coverslip. The worms were examined using a Zeiss LSM 510 META system connected to an inverted Zeiss Axiovert 200 microscope. The z-stacks were projected in 360° using 32 or 64 steps and then exported as full resolution images in avi or mov format using the in-microscope software LSM 510 ConfoCor2 Combination, version 3.2. These movies were then used to determine the degree of twisting in the isthmus using video editing software (Sorenson squeeze, trial version). Worms carrying pMH86 was examined using BioRad radiance 2000 setup. The z-stackswas projected in 360° in 36 steps using the in-microscope software Laser Sharp 2000 and thereafter scored using the image processing program ImageJ. "180° twist" means that the distal ends of the M2 neurons would have to be rotated by 180° in order to be parallel with the cell bodies from which they originate.

### Dissection of pharynges

Worms to be dissected were held in place on dried agarose pads (2% in H_2_O) covered with mineral oil. The dissection was done under a Leica stereomicroscope using a tungsten (0.5 mm) dissecting needle. Isolated pharynges were transferred to a drop of M9 on an agarose pad (2% in M9) and allowed to relax by swirling the liquid before applying a coverslip, and examined under the microscope.

### Feeding and pumping rate assays

For the feeding assay, L4 larvae of N2, *mig-4*, *dig-1 *or *mnm-4 *genotypes were placed on nematode growth medium (NGM) plates covered with a 500:1 (vol:vol) mixture of OP50 bacteria and Fluoresbrites Multifluorescent microspheres, 0.2 μm (Polyscience, Inc.), essentially as previously described [39]. Briefly, the mixture of bacteria and beads was added to the culture plates 20 h before the worms were allowed to feed for 30 min and then transferred to eppendorf tubes with 1 ml of M9 plus levamisole (1 mM). The tubes were centrifuged at 2000 *g *for 2 min at room temperature and the supernatant removed, leaving approximately 30 μl in which to resuspend the worms for transfer to a freshly made agarose pad (2% agarose in M9 buffer). The worms were covered with a cover slip and the beads observed by epifluorescence microscopy at 1 000× magnification and counted.

To investigate the transfer rate of bacteria into the intestine, batches of 15–20 worms were placed on NGM plates seeded with OP50 and beads (as above but using 1 000:1 mixtures) and allowed to explore for 2 minutes. The worms were thereafter mounted on agarose pad (2% agarose in M9 buffer) in a drop of 100 mM levamisol, covered with a cover slip and scored for the presence beads in the intestine and the pharynx using epifluorescence microscopy at 400× magnification. Worms that after 2 minutes were outside the bacteria/bead lawn or still remained where they were initially placed were excluded from the analysis.

To determine the pumping rate, eight worms of each genotype were placed onan NGM plate seeded with OP50 and observed using a stereomicroscope for ten consecutive seconds during which the number of pumps was recorded.

Starved L1s: Hermaphrodites were bleached over night on empty NGM plates. Resulting L1 larvae were transferred to 1,5 ml microcentrifuge tubes with M9 and kept at RT for ~72 hours. The tubes where thereafter emptied on unseeded NGM plates and pumping rate was scored in stereomicroscope. Only non-necrotic larvae were scored.

### Electropharyngeograms

An electrophysiology setup was modified essentially as described in a published protocol to record electropharyngeograms [13]. To encourage pumping, the worms were kept in Dent's saline containing 1 μM serotonin [13]. Several dozen pharyngeal pumps were recorded from at least ten worms for each studied genotype.

### Electron Microscopy

Specimens were immersion fixed using buffered aldehydes and then osmium tetroxide as described previously [40]. Three or four animals were aligned within agar blocks then embedded in plastic resin and sectioned together. Thin cross sections were collected on slot grids, post-stained with uranyl acetate and lead citrate, then examined with a Philips CM10 electron microscope. For embryo studies, a laserhole protocol was instead used to permit fixative to pass the eggshell [41].

For close examination of basal lamina, wild type animals were fixed by high pressure freezing and freeze substitution [42] before embedment into resin. Thin sections were again post-stained and examined in a Philips CM10 electron microscope. Additional wild type animals were immersion fixed and exposed to microwave energy using a Pelco Biowave oven to help aldehydefixatives and osmium fixatives to cross the cuticle efficiently. Fixed animals were then mounted in agar blocks and embedded in plastic resin following standard protocols for thin sectioning.

## Authors' contributions

CA carried out most of the experiments and helped in the writing of the manuscript. MR performed the electropharyngeograms. DHH provided the electron microscopy results, and helped in interpretations of the results and the writing of the manuscript. MP helped in experimental design and interpretation and took the main responsibility for writing the article.

## Supplementary Material

Additional File 1 M2 neurons in wild-type. This movie shows a confocal microscopy reconstruction of the M2 neuron trajectories in a wild-type animal expressing the *etIs2 *transgene. In wild-type the M2 neurons are mostly straight and parallel throughout their entire trajectory. Anterior is at bottom. Click here for file

Additional File 2 M2 neurons in *mnm-4 *mutant. This movie shows a confocal microscopy reconstruction of the M2 neuron trajectories in an *mnm-4 *mutant animal expressing the *etIs2 *transgene. In the *mnm-4 *mutant, pharyngeal twisting is evidenced by the double-helix pattern of the M2 neuron processes. Anterior is at bottom. Click here for file
